# Audit of Postoperative Complications and Conversion Rate in Laparoscopic Cholecystectomy

**DOI:** 10.7759/cureus.91249

**Published:** 2025-08-29

**Authors:** Hassam Zaka Khan, Taha Ahmed Tarin, Saadia Kanwal, Bilawal Ali, Hafiz Muhammad Arbaz, Chaudhary Adeel Ahmad, Rabia Khan

**Affiliations:** 1 Kidney Transplant, Pakistan Kidney and Liver Institute, Lahore, PAK; 2 Internal Medicine, Combined Military Hospital (CMH) Lahore Medical College and Institute of Dentistry, Lahore, PAK; 3 General Surgery, Shalamar Hospital, Lahore, PAK; 4 Internal Medicine, District Headquarters (DHQ) Teaching Hospital, Dera Ghazi Khan, PAK; 5 General Surgery, Mayo Hospital, Lahore, PAK; 6 General Surgery, Benazir Bhutto Hospital, Rawalpindi, PAK

**Keywords:** conversion rate, emergency surgery, gallbladder disease, laparoscopic cholecystectomy, postoperative complications

## Abstract

Background

Laparoscopic cholecystectomy (LC) is the standard procedure for the management of symptomatic gallbladder disease, offering numerous benefits such as reduced pain and faster recovery compared to open surgery.

Objective

This study aimed to determine the incidence and types of postoperative complications and assess factors associated with conversion to open surgery in patients undergoing LC.

Methods

This is a retrospective cohort study, conducted at Shalamar Hospital, Lahore, Pakistan, from June 2023 to June 2025. A total of 355 patients who underwent the procedure were included in the study using a non-probability consecutive sampling technique. Data were collected retrospectively from the hospital’s surgical database and patient medical records. Information on patient demographics, comorbidities, surgical details, postoperative complications, and conversion rates was extracted for analysis.

Results

Among 355 patients (mean age: 48.3 ± 12.7 years; 67% women), 43 patients (12%) developed postoperative complications. The most common were surgical site infection (5%) and bile duct injury (2.5%). The overall conversion rate was 3.4%, higher in emergency (9.3%) compared to elective (2.2%) procedures. Logistic regression showed significant associations between postoperative complications and obesity (odds ratio {OR}, 3.5; 95% confidence interval {CI}, 1.5-8.2; p = 0.002), emergency surgery (OR, 2.1; 95% CI, 1.2-3.6; p = 0.008), and diabetes (OR, 2.3; 95% CI, 1.1-4.8; p = 0.03). Conversion was significantly associated with acute cholecystitis (OR, 4.1; p = 0.002), previous abdominal surgery (OR, 4.2; p = 0.01), obesity (OR, 3.3; p = 0.004), and emergency surgery (OR, 2.3; p = 0.04).

Conclusion

Laparoscopic cholecystectomy is a safe and effective procedure with low complication and conversion rates. Obesity, diabetes, and emergency presentation were significantly associated with adverse outcomes, underscoring the need for careful patient selection, preoperative optimization, and timely elective surgery. These associations should be confirmed through future prospective studies.

## Introduction

Laparoscopic cholecystectomy (LC), introduced in the early 1990s, has become the preferred surgical technique for the treatment of symptomatic gallbladder disease. Its noninvasive trait has transformed the way gallbladder surgery is treated and has been named to benefit the patient, i.e., less pain after surgery, less hospitalization, and faster healing, when compared to the traditional open tourniquet cholecystectomy [1]. The procedure has become the standard of care among the majority of patients suffering from gallbladder stones with chronic cholecystitis or even in treating acute cholecystitis, especially when using elective cases. The success of laparoscopic cholecystectomy has predetermined its standardization, but one must remember that, similar to any surgical technique, LC is accompanied by risks [2]. However, the laparoscopic approach is not without complications, although it generally has fewer adverse outcomes compared to the open approach [3]. The most frequent postoperative complications include biliary injury, hemorrhage, infection, and anesthesia-related complications. Among these, biliary duct injury is the most serious, with potential long-term consequences such as bile leakage, strictures, and the need for further surgical interventions [4]. Management often requires a multidisciplinary approach, and in severe cases, patients may require reoperations or even liver transplantation. Fortunately, the incidence of such complications has decreased significantly with advances in surgical expertise and techniques; nonetheless, it remains a primary concern in laparoscopic cholecystectomy (LC) [5].

Several factors contribute to the risk of complications, with surgeon experience being among the most critical [6]. Studies have shown that complication rates decline with increasing surgical volume and expertise, highlighting the importance of the surgeon’s skill level and the institution’s experience [7]. There is also a group of factors relating to the patient that might complicate the undertaking of the operation and enhance the chance of complications; it could include obesity, old age, diabetes mellitus, and the presence of past surgeries on the abdominal cavity [8]. Obesity, especially, gives such a problem to laparoscopy as the inability to see the operative field, and the time spent as a result of that obesity takes a long time, with the risk of injuring the neighboring structures [9]. The conversion of laparoscopic to an open cholecystectomy, in addition to postoperative complications, is also a vital indicator of its safety [10]. Open surgery conversion is one of the most common and is normally done when the laparoscopic methods are suspected no more to be safe, most likely because of the technical challenge that has been met during the surgery [11]. The complications that often require conversion include bleeding, bile duct damage, indeterminate anatomy, or inability to see major anatomy such as the cystic duct or cystic artery [12]. Though, over the years, the conversion rates have declined with better skills and technologies in laparoscopy, such rates still form a safety net to patients when attempts at laparoscopy are not successful [13]. Conversion rates ranging from 0.5% to 10% have been reported in studies, compared to instances of acute cholecystitis or in individuals with an anatomical variation thereof in the form of a previous abdominal surgery or a difficult gallbladder [14]. There are several factors that determine the decision to switch to open surgery. The safety of the patient is usually of primary concern to surgeons, where conversion is an option in case of the possibility of causing severe damage to critical structures [15].

Objective

This study aimed to determine the incidence and types of postoperative complications and assess factors associated with conversion to open surgery in patients undergoing LC.

## Materials and methods

Methodology

This is a retrospective cohort study, conducted at Shalamar Hospital, Lahore, Pakistan, from June 2023 to June 2025. A total of 355 patients who underwent the procedure were included in the study using a non-probability consecutive sampling technique.

Inclusion and exclusion criteria

The inclusion criteria for the study were adults aged 18-80 years who underwent elective or emergency laparoscopic cholecystectomy for symptomatic cholelithiasis or acute cholecystitis. Eligible patients had a preoperative diagnosis of gallstones, biliary colic, or acute cholecystitis and underwent laparoscopic cholecystectomy as the primary procedure, with or without other associated surgical interventions.

Patients were excluded if they underwent cholecystectomy for non-biliary causes such as cancer, had major contraindications to laparoscopic surgery including severe cardiopulmonary disease or pregnancy, or experienced intraoperative conversion to open surgery for reasons unrelated to biliary pathology. Patients with other biliary pathologies, such as choledocholithiasis, cholangitis, biliary strictures, or malignancy, were explicitly excluded.

Data collection

Data were collected retrospectively from the hospital’s surgical database and patient medical records. Information on patient demographics, comorbidities, surgical details, postoperative complications, and conversion rates was extracted for analysis. The main variables were the age, sex, body mass index (BMI), comorbid illnesses such as diabetes or hypertension, and the indication of surgery. Perioperative surgical information on the length of procedure, the nature of the cholecystectomy performed (elective or emergency), and intraoperative problems was also documented. The postoperative complication was divided into biliary injuries, bleeding, infection, and other complications. Conversion rate data were obtained with the reasons why conversion to open surgery occurred, following difficulty imaging key structures or intraoperative complications, among other reasons. None of the patients in our cohort died during hospitalization or within the early postoperative period. This study had primary outcome measures of the frequency, and they were the type of postoperative complications, such as bile duct injury, hemorrhage, infection, and other surgery-related complications. The conversion rate, which is the necessity of the conversion of laparoscopic to open cholecystectomy, was also evaluated. Further, the purpose of the study was to assess how the demographics of patients, comorbidities, and surgical factors relate to the presence of complications and risks of conversion. This was in order to establish possible risk factors that may assist in forecasting bad occurrences and customize preventative strategies.

Statistical analysis

The data were analyzed using SPSS version 26.0 (IBM Corp., Armonk, NY). Descriptive statistics were calculated to summarize demographic characteristics, surgical details, and complication rates. Categorical variables such as gender, complications, and conversion rates were expressed as frequencies and percentages, while continuous variables such as age and BMI were presented as means ± standard deviation. Multivariate logistic regression analysis was performed to assess independent risk factors for postoperative complications and conversion to open surgery. A p-value of ≤0.05 was considered statistically significant.

## Results

Data were collected from 355 patients, with a mean age of 48.3 ± 12.7 years. Women represented the majority (238, 67%) of the cohort, with men comprising 117 (33%). Regarding body mass index (BMI), 255 (72%) of patients were classified as having a normal BMI, 52 (15%) were overweight, and 47 (13%) were obese. Comorbidities were common, with 82 (23%) of patients having hypertension, 64 (18%) having diabetes mellitus, and 35 (10%) having cardiovascular diseases. The majority of procedures were elective (312, 88%), and the average surgical duration was 75 ± 15 minutes for elective surgeries and 85 ± 20 minutes for emergency cases. Intraoperative difficulties were encountered in 53 (15%) of cases, with the most common issues being difficulty identifying critical structures and severe inflammation/adhesions (Table 1).

**Table 1:** Patient Demographics and Baseline Data Data are represented as mean ± SD or percentages (%) SD, standard deviation; BMI, body mass index

Demographic Characteristic	Value (n = 355)
Age (Mean ± SD)	48.3 ± 12.7 Years
Gender	
Female	67% (238)
Male	33% (117)
BMI	
Normal (18.5-24.9)	72% (255)
Overweight (25-29.9)	15% (53)
Obese (>30)	13% (47)
Comorbidities	
Hypertension	23% (82)
Diabetes Mellitus	18% (64)
Cardiovascular Diseases	10% (35)
Type of Surgery	
Elective	88% (312)
Emergency	12% (43)
Surgical Duration (Mean ± SD)	75 ± 15 Minutes (Elective); 85 ± 20 Minutes (Emergency)
Intraoperative Difficulties	15% (53)
Difficulty Identifying Cystic Duct/Artery	7% (25)
Severe Inflammation/Adhesions	5% (18)

Postoperative complications were observed in 43 (12%) patients. The most common complications were surgical site infections (18, 5%), followed by bile duct injury (nine, 2.5%) and hemorrhage (seven, 2%). Gastrointestinal issues such as ileus and bowel injury occurred in six (1.7%) patients, while other complications such as deep vein thrombosis (DVT) and urinary retention accounted for seven (1.8%). The overall conversion rate was 12 (3.4%), with elective surgeries having a conversion rate of eight (2.2%), while emergency surgeries had a higher rate of four (9.3%). The main reasons for conversion included difficulty in identifying critical structures (seven, 58%), bleeding (four, 33%), and unrecognized bile duct injury (one, 8%) (Table 2).

**Table 2:** Postoperative Complications Values were expressed as percentages (%) DVT: deep vein thrombosis

Complication Type	Frequency (%)	Number of Cases (n = 355)
Surgical Site Infection	5%	18
Bile Duct Injury	2.5%	9
Hemorrhage	2%	7
Gastrointestinal Issues	1.7%	6
Miscellaneous (DVT, Urinary Retention, etc.)	1.8%	7
Conversion Characteristic		
Overall Conversion Rate	3.4%	12
Elective Surgery	2.2%	8
Emergency Surgery	9.3%	4
Reasons for Conversion		
Difficulty in Identifying Critical Structures	58%	7
Bleeding	33%	4
Unrecognized Bile Duct Injury	8%	1

**Figure 1:**
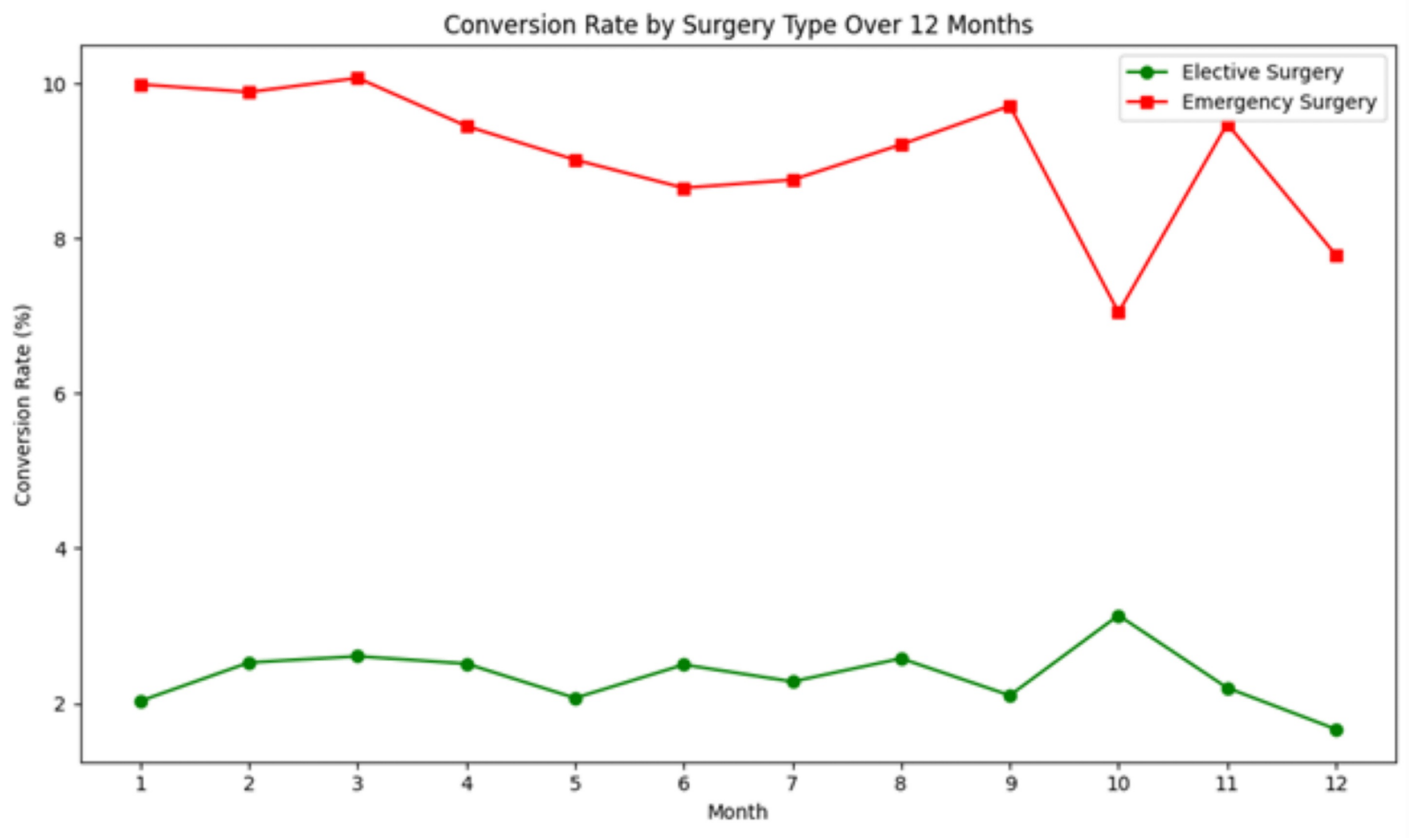
Conversion Rates for Elective and Emergency Surgeries Over 12 Months It shows that emergency surgeries experience more fluctuation in conversion rates compared to the relatively stable rates of elective surgeries

Multivariate logistic regression revealed that obesity (BMI: >30) was the factor most strongly associated with postoperative complications, with an odds ratio (OR) of 3.5 (95% confidence interval {CI}: 1.5-8.2; p = 0.002). Emergency surgery was also significantly associated, with an OR of 2.1 (95% CI: 1.2-3.6; p = 0.008). Diabetes mellitus increased the risk of complications with an OR of 2.3 (95% CI: 1.1-4.8; p = 0.03). Conversion to open surgery was significantly associated with acute cholecystitis (OR = 4.1; p = 0.002), previous abdominal surgery (OR = 4.2; p = 0.01), obesity (OR = 3.3; p = 0.004), and emergency surgery (OR = 2.3; p = 0.04) (Table 3 and Figure 1).

**Table 3:** Risk Factors for Postoperative Complications Odds ratios (ORs) with 95% confidence intervals (CI) are reported. Significance level set at p < 0.05 BMI: body mass index

Risk Factor	Odds Ratio (OR)	95% Confidence Interval (CI)	P-value
Obesity (BMI: >30)	3.5	1.5-8.2	0.002
Emergency Surgery	2.1	1.2-3.6	0.008
Diabetes Mellitus	2.3	1.1-4.8	0.03
Risk Factors for Conversion to Open Surgery			
Acute Cholecystitis	4.1	1.6-10.4	0.002
Previous Abdominal Surgery	4.2	1.3-13.1	0.01
Obesity (BMI: >30)	3.3	1.4-7.9	0.004
Emergency Surgery	2.3	1.1-5.4	0.04

The average length of hospital stay for elective surgeries was 1.8 ± 0.7 days, whereas emergency surgeries required a longer stay of 3.2 ± 1.1 days. The postoperative readmission rate was 11 (3%), with infection being the primary cause of readmission in seven (64%) of cases, followed by bile duct injury in four (36%) of cases (Table 4).

**Table 4:** Length of Hospital Stay and Readmission Rates Data are represented as mean ± SD or percentages (%) SD: standard deviation

Characteristic	Value (n = 355)
Length of Stay (Mean ± SD)	1.8 ± 0.7 Days (Elective); 3.2 ± 1.1 Days (Emergency)
Postoperative Readmission Rate	3% (n = 11)
Reasons for Readmission	
Infection	64% (Seven Cases)
Bile Duct Injury	36% (Four Cases)

## Discussion

Laparoscopic cholecystectomy (LC) remains the gold standard for the management of symptomatic cholelithiasis and other gallbladder diseases, largely due to its minimally invasive nature, shorter recovery times, and reduced postoperative pain compared to open surgery. Problems and the necessity of conversion to open surgery have been noted, even with evidence of high success rates and widely used techniques, which may pose difficulties. The aim of the study was to assess the complication rate after the surgery, to report the factors that impact the conversion rate, and to assess risk factors of the complications and conversion. The total postoperative complication rate in this study was 12%, which is comparable to other reports documenting rates between 5% and 20% [16]. Of the complications, the most frequent ones were surgical site infections (5%) and bile duct injuries (2.5%).

Injuries to the bile ducts should be of high concern due to the possibility of imposing serious long-term effects on the individuals based on the development of a bile leak and strictures, which can necessitate further surgical repair or even necessitate liver transplantation. Even though the outcome of this study with respect to the incidence of the bile duct injury is in line with the literature, it is one of the most dreaded sequelae of LC [17]. The rate of incurring complications was markedly elevated among patients who received emergency procedures when compared to those with elective procedures. Such a finding is congruent with the literature, which indicates that the instability of the inflammatory process and adhesions occurring in cases of acute cholecystitis may turn treatment via laparoscopic access complicated and potentially harmful [18]. Findings of this study support that diabetes mellitus is associated with a higher risk of surgical infections as a result of interference in immune activity and the healing of wounds caused by the condition [19]. The total conversion on the same study was 3.4-fold, not very off range considering the range usually found in the study literature (2-10-fold). Conversion to open surgery is sometimes seen as a safety procedure, and its advantage is that the conversion rate in the current study was not very high. Conversion had more to do with anatomical challenges (58%), bleeding (33%), and missed injury to the bile duct (8%) [20].

Conversion rate in the emergency cases was considerably higher in comparison to elective surgeries (9.3% versus 2.2%), and it is probably a result of acute cholecystitis, as well as the development of pronounced adhesions, which heighten the propensity of conversion. On the same note, the identification of a previous abdominal operation was a major risk factor for conversion since adhesions might block the field and make the surgery intricate. The induction of surgeons is aimed at ensuring maximum care of the patient, and failure to complete a procedure due to patient safety is not the goal of surgeons but to prevent injury to vital structures, most importantly the bile ducts [21]. Multivariate analysis identified several variables that were significantly associated with postoperative complications and conversion, including obesity, diabetes, and emergency surgery. Obesity was found to be an important predictor of both conversion and postoperative complications, in which obese patients were reported to have a 3.5-fold strong association with overall postoperative complications [22]. This is in harmony with the literature at hand, which indicates that obesity is a challenge to laparoscopic surgery because of bad visualization, inability to get into the gallbladder, and the delay of operating time [23]. This observation is consistent with those made in other reports that showed that patients having postoperative complications have a longer length of stay in hospitals. In this study, the readmission rate stands at 3%, which is low enough to indicate that the majority of patients have had positive postoperative results [24].

In summary, laparoscopic cholecystectomy remains a safe and effective treatment for gallbladder disease, with relatively low rates of complications and conversion. However, outcomes are less favorable in patients with obesity and diabetes and those undergoing emergency surgery. These findings, consistent with prior studies, highlight the importance of early elective intervention and preoperative optimization in high-risk patients. Importantly, the identified associations should be interpreted with caution, as retrospective analyses cannot establish causality. Future prospective multicenter studies are necessary to validate these observations and provide stronger evidence for clinical guidelines.

This study has several limitations that must be acknowledged. Firstly, its retrospective design introduces inherent biases, including the risk of incomplete or inaccurate medical records, and limits the ability to establish causal relationships. The identified risk factors should therefore be interpreted as associations rather than definitive predictors of postoperative complications or conversion to open surgery. Secondly, being a single-center study, the findings may not be generalizable to other healthcare settings with different surgical expertise, patient demographics, or resources. Thirdly, the absence of prospective follow-up restricted our ability to assess long-term outcomes, such as late bile duct strictures or recurrent biliary events. Despite these limitations, the relatively large sample size and systematic data analysis strengthen the reliability of our observations. Future prospective multicenter studies are warranted to confirm these associations and provide stronger evidence regarding causality.

## Conclusions

It is concluded that laparoscopic cholecystectomy remains a safe and effective procedure for the treatment of symptomatic gallbladder disease, with low complication and conversion rates. The study found a 12% incidence of postoperative complications, with surgical site infections and bile duct injuries being the most common. The overall conversion rate to open surgery was 3.4%, with higher rates observed in emergency surgeries, particularly those involving acute cholecystitis. Factors such as obesity, diabetes, and emergency presentation were significantly associated with increased complications and conversion. These findings underscore the need for careful patient selection, early elective surgery, and preoperative optimization while highlighting the importance of future prospective studies to confirm these associations.
